# Expression of Inflammatory Markers in Ascending Thoracic Aorta in Patients with Obstructive Sleep Apnea

**DOI:** 10.3390/ijms27146333

**Published:** 2026-07-16

**Authors:** Wioletta Olejarz, Ewa Migacz, Andrzej Łoś, Katarzyna Bednarek-Rajewska, Andrzej Kluk, Anna M. Czarnecka, Wojciech Kukwa

**Affiliations:** 1 Department of Biochemistry and Pharmacogenomics, Faculty of Pharmacy, Medical University of Warsaw, 02-091 Warsaw, Poland; wioletta.olejarz@wum.edu.pl; 2 Centre for Preclinical Research, Medical University of Warsaw, 02-097 Warsaw, Poland; 3 Department of Otorhinolaryngology, Faculty of Medicine and Dentistry, Medical University of Warsaw, 02-091 Warsaw, Poland; ewamigacz@gmail.com; 4 Department of Cardiac and Vascular Surgery, Medical University of Gdansk, 80-214 Gdansk, Poland; andrzej.los@gumed.edu.pl; 5 Department of Clinical Pathology, Poznan University of Medical Sciences, 60-355 Poznan, Poland; szlapus@icloud.com (K.B.-R.); akluk@interia.pl (A.K.); 6 Department of Outpatient Chemotherapy, Maria Sklodowska—Curie National Research Institute of Oncology, 02-781 Warsaw, Poland; anna.czarnecka@gmail.com; 7 Department of Experimental Pharmacology, Mossakowski Research Institute, Polish Academy of Sciences, 02-106 Warsaw, Poland

**Keywords:** obstructive sleep apnea, atherosclerosis, ICAM-1, VCAM-1, TNF-α, IL-6

## Abstract

Obstructive sleep apnea (OSA)-induced hypoxia modulates inflammatory mediators associated with atherosclerosis and cardiovascular diseases. ICAM-1, VCAM-1, TNF-α and IL-6 are involved in atherogenesis, but their expression in the aortic walls of OSA patients remains unknown. This study aimed to determine the relationship between OSA severity and inflammatory markers expression in aortic tissue from patients undergoing coronary artery bypass grafting (CABG). This study included 46 patients who underwent CABG. OSA severity was assessed using the WatchPAT™ home sleep apnea test, classifying patients into control and mild (0 < AHI < 15) and moderate-to-severe (AHI ≥ 15) OSA groups. Aortic wall samples were collected intraoperatively, and ICAM-1, VCAM-1, TNF-α and IL-6 expression was evaluated using immunohistochemistry. Statistical analysis compared protein expression across OSA severity groups. Compared with control and mild OSA, in aorta of the moderate-to-severe OSA group, significant differences for ICAM-1 (*p* = 0.001) and VCAM-1 (*p* = 0.006) expression were demonstrated. This study provides novel evidence of significantly increased ICAM-1 and VCAM-1 expression in the aortic walls of patients with moderate-to-severe OSA. No statistically significant differences were observed for TNF-α and IL-6. Our findings offer a rationale for integrating vascular inflammation markers into cardiovascular risk stratification in OSA. They also support the concept of OSA as a systemic inflammatory disorder, with tangible effects on large-vessel morphology.

## 1. Introduction

Atherosclerosis is a chronic inflammatory disease characterized by lipid accumulation, endothelial dysfunction, and progressive narrowing of blood vessels, ultimately leading to ischemic events such as myocardial infarction and stroke [1]. Inflammation within the aortic wall can be caused by infectious or immune mechanisms, leading to complications such as the formation, rupture, or dissection of aneurysms [2,3]. Destabilization of atherosclerotic plaques—marked by increased immune cell infiltration and cytokine activity—is a critical event in the pathogenesis of acute cardiovascular episodes [4]. Beyond conventional risk factors, emerging evidence suggests that disturbed sleep, particularly obstructive sleep apnea (OSA), contributes to the development and progression of atherosclerosis [5,6]. OSA is characterized by recurrent collapse of the upper airways during sleep, leading to intermittent hypoxia, sleep fragmentation, and increased sympathetic activity [7]. Several studies have demonstrated a link between OSA severity and subclinical systemic atherosclerosis, independent of traditional cardiovascular risk factors [2,3,4]. OSA-induced hypoxia initiates a cascade of pathophysiological changes, including activation of inflammatory pathways, oxidative stress, and vascular dysfunction [5].

**Figure 1:**
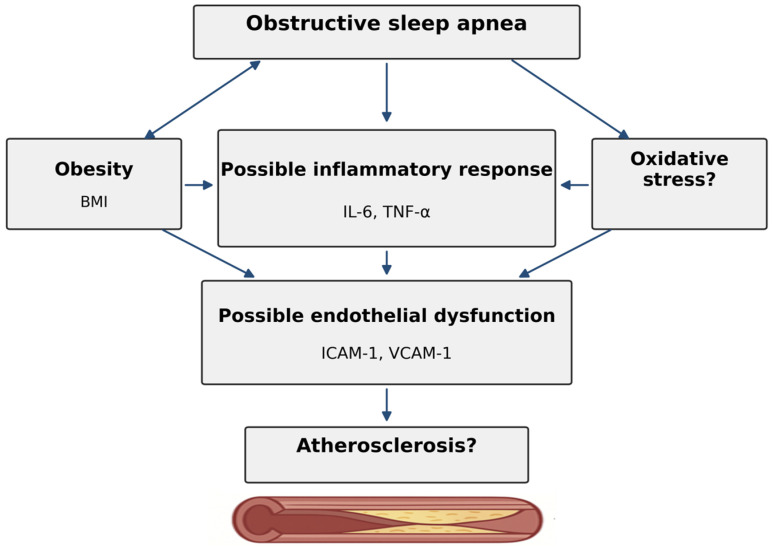
Are inflammatory markers ICAM-1, VCAM-1, TNF-α and IL-6 in the ascending thoracic aorta associated with OSA severity?

Chronic intermittent hypoxia (CIH; hypoxia–reoxygenation) in patients with OSA induces sympathetic activation, ROS generation, and redox signaling, leading to activation of NF-κB (systemically and in immune cells) and subsequent production of TNF-α production. Resultant endothelial activation (ICAM-1/VCAM-1 expression) and innate immune and adipose inflammation (TNF-α/IL-6) promote leukocyte adhesion, endothelial dysfunction, and atherogenesis. In general, OSA is associated with elevated circulating levels of inflammatory markers, including intercellular adhesion molecule-1 (ICAM-1), vascular cell adhesion molecule-1 (VCAM-1), tumor necrosis factor-alpha (TNF-α) and interleukin-6 (IL-6) [6,7,8]. To date, little is known about the expression of these proteins in the vascular wall itself, the primary site of the development of atherosclerotic lesions. Even less is known about OSA-induced gene expression in the aortic wall. Therefore, since these proteins are involved in a single pathophysiological mechanism of atherogenesis, this study aimed to assess whether expression levels of ICAM-1, VCAM-1, TNF-α, and IL-6 in the ascending thoracic aorta are associated with OSA severity (Figure 1).

## 2. Results

The mean age was 67.4 ± 8.3 years. The control group (n = 4) included patients with Apnea–Hypopnea Index (AHI) < 5 events/h. There were 18 patients (39.1%) with mild OSA, 11 (23.9%) with moderate OSA, and 13 (28%) with severe OSA (Table 1). Patients with moderate-to-severe OSA had higher BMI, HbA1c, total cholesterol, LDL, and CRP than those with control or mild OSA (*p* < 0.05). Data regarding sleep study results are also shown in Table 1.

**Figure 2:**
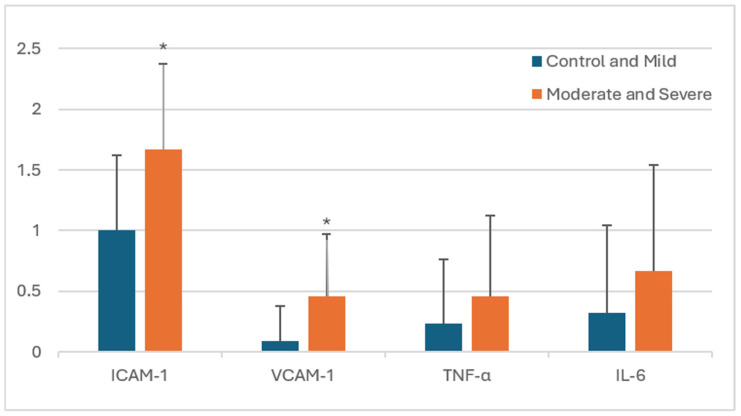
Levels of inflammatory markers measured in aortic wall of two groups of patients with obstructive sleep apnea: control + mild (n = 22), and moderate + severe (n = 24). * *p* < 0.05. On the Y axis: the number of positive cells categorized using the following 4-tiered classification system: 0, negative reaction (less than 10% of stained cells); 1, low expression (11–50% of cells stained positive); 2, intermediate expression (51–75% of cells stained positive); 3, high expression (over 75% of cells stained positive). Bars represent mean expression scores ± SD.

**Figure 3:**
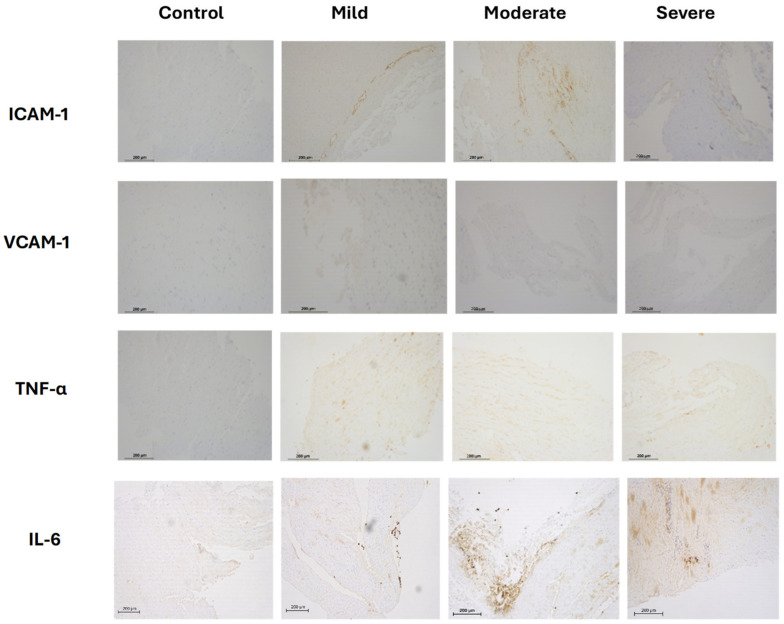
Immunohistochemical analysis of ICAM-1, VCAM-1, TNF-α, and IL-6 in the aortic wall in patients undergoing coronary artery bypass grafting. Immunohistochemistry was performed using rabbit or mouse antibodies and an isotype-matched IgG control. Representative images from each group (control, mild, moderate, and severe OSA) are presented. Original magnification: Å~200. Scale bar = 200 μm.

Immunohistochemistry demonstrated significantly increased expression of ICAM-1 (*p* = 0.001) and VCAM-1 (*p* = 0.006) in the aorta of the moderate-to-severe OSA group compared with the control and mild OSA group. No statistically significant differences were observed for TNF-α and IL-6 (Figure 2, Table 2). The strongest immunohistochemical reaction was observed in stromal inflammatory cells, in cells morphologically resembling lymphocytes and monocytes. The expression of these markers was associated with OSA severity (Figure 2 and Figure 3). A positive immunohistochemical reaction in endothelial cells was also observed. Figure 2 presents selected data from Table 2 in graphical form, focusing on differences in inflammatory marker expression between the analyzed groups.

Adjustment of the analysis for BMI attenuated the significance of ICAM-1, although the association remained statistically significant (*p* = 0.034). BMI was also associated with the ordinal outcome (*p* = 0.039). For VCAM-1, TNF-α, and IL-6, however, inclusion of BMI did not significantly improve our tested model fit in likelihood ratio (*p* = 0.633, *p* = 0.972, and *p* = 0.494, respectively), suggesting that BMI adjustment is not warranted for these markers. Adjustment for BMI attenuated but did not abolish the association for ICAM-1. For VCAM-1, the association with OSA severity remained independent of BMI. TNF-α and IL-6 did not show statistically significant associations with OSA severity.

## 3. Discussion

Our study is unique in that it directly examined aortic wall tissue collected intraoperatively during CABG, allowing us for the first time to demonstrate significantly increased expression of the proinflammatory markers ICAM-1 and VCAM-1 in patients with moderate-to-severe OSA, while no statistically significant differences were observed for TNF-α and IL-6, thereby providing evidence linking OSA severity with vascular inflammation. Our findings built on previous work showed elevated local levels of these markers in OSA and further emphasize the role of localized vascular inflammation in the atherogenic process. These inflammatory markers were explicitly selected due to the growing interest in their role in cardiovascular disease. They have been implicated in mediating inflammatory processes, facilitating leukocyte recruitment and activation at sites of vascular injury, and contributing to plaque development and destabilization. Until now, we found increased expression of CD40 and CD40L receptors, MCP-1, and MMP-9 in atherosclerotic plaque, proportional to OSA severity [9]. In our second study, we described increased expression of TLR2, TLR4, TLR9, and RAGE in carotid plaques of patients with moderate-to-severe OSA compared with controls without OSA and those with mild OSA, suggesting that TLR- and RAGE-mediated pathways may contribute to OSA-related atherogenesis [10]. We also showed that patients with moderate or severe OSA had higher expression of S100A8 and S100A9 in aortic tissue than the control group and the mild OSA group, providing new evidence that S100A8 and S100A9 may play a role in vascular inflammation and atherosclerosis associated with OSA [11]. This research is our second study (and, to our knowledge, the second in the literature) examining proatherogenic factors in whole aortic walls in patients with OSA. This allowed us to quantitatively measure ICAM-1, VCAM-1, TNF-α, and IL-6 in the ascending thoracic aorta.

Serum levels of inflammatory markers, including ICAM, VCAM, IL-6, and TNF-α, were previously analyzed and shown to be higher in OSA patients than in control subjects [6]. Fiedorczuk et al. demonstrated that serum and plasma biomarkers CRP, S100B, IL-6, TNF-α, and IL-8 are elevated in patients with OSA and correlate positively with disease severity [12]. Also, Arnardottir et al. confirmed that the severity of OSA independently predicts IL-6 and CRP levels. However, this relationship was observed only in obese individuals [13], underscoring the importance of these markers in assessing cardiovascular risk. OSA is consistently associated with activation of endothelial adhesion pathways, with the human evidence appearing stronger for ICAM-1 than for VCAM-1. A recent meta-analysis reported elevated circulating ICAM-1 in adults with OSA and supported its potential utility as a biomarker of OSA-related cardiovascular risk [14], while a parallel meta-analysis linked higher circulating VCAM-1 with OSA and endothelial dysfunction [7]. Treatment-response data further favor ICAM-1—recent CPAP efficacy meta-analysis showed a significant reduction in circulating ICAM-1 (and E-selectin) after treatment, whereas VCAM-1 did not significantly improve after CPAP [15]. Likewise, in a longitudinal cohort of patients evaluated for OSA, higher baseline ICAM-1 levels were associated with cardiovascular events over 8 years, whereas VCAM-1 was not significantly predictive for such events in the same analysis. To strengthen the mechanistic interpretation and better separate the specific contribution of intermittent hypoxia (IH) from confounders such as obesity or dyslipidemia, animal models were developed. In rats, chronic intermittent hypoxia increased ICAM-1 expression in an ischemia–reperfusion model, and a direct comparison of chronic intermittent versus chronic continuous hypoxia showed that intermittent hypoxia exerted a greater effect on endothelial-cell adhesion, vasoconstrictive/procoagulant changes, endothelial dysfunction, and myocardial contractility impairment. In parallel, in a mouse model, it was demonstrated that IH induces vascular remodeling and atherogenesis, including increased transendothelial LDL and monocyte passage and vascular injury. Thus, rodent IH studies confirm that at least part of the ICAM/VCAM expression observed in OSA reflects a direct biological effect of intermittent hypoxia itself, rather than the influence of accompanying metabolic comorbidities [7,14,15,16,17,18,19].

Our observations align with intermittent hypoxia—characteristic of OSA—that triggers oxidative stress and inflammatory cascades. At the center of this cascade are cell adhesion molecules such as ICAM-1 and VCAM-1, which mediate leukocyte adhesion and transendothelial migration—critical early steps in atherogenesis [20,21]. ICAM-1 (CD54) is expressed on the aortic endothelium and can be expressed and upregulated on vascular smooth muscle cells (VSMCs) under inflammatory stimulation [22]. OSA-induced hypoxia might result in cardiovascular disease due to increased expression of ICAM-1 and VCAM-1 as early change at the vascular wall level [23]. VCAM-1 (CD106) is strongly expressed on the activated aortic endothelium in atherogenic/inflammatory states, also detectable in the medial layer of VSMC compartment [24]. VCAM-1 was also demonstrable in aortic tissues from atherosclerotic patients and its expression was shown to correlate with disease burden [25]. Qianwen Lv et al. demonstrated that increased levels of VCAM-1 are associated with a higher incidence of coronary artery disease in adults with moderate-to-severe OSA [26]. TNF-α is a cytokine produced locally in inflamed aortic tissue, primarily by infiltrating macrophages and other immune cells, and can act on VSMCs and endothelium to amplify inflammation and remodeling. TNF-α significantly contributes to atherosclerosis by triggering the expression of VCAM-1 and ICAM-1 [27,28,29]. IL-6 can be produced by aortic endothelial cells and aortic VSMCs and acts in an autocrine and paracrine manner to promote vascular inflammation and dysfunction [30]. IL-6 can also drive oxidative stress signaling in aortic VSMCs via AT1R upregulation with downstream ROS signaling, linking IL-6 activity to endothelial and vascular dysfunction pathways [31].

VCAM-1 and ICAM-1 contribute to atherogenesis by facilitating monocyte accumulation in the arterial intima, which is associated with coronary artery disease (CAD) in OSA [26,32]. ICAM-1 and MCP-1 coordinate monocyte recruitment and infiltration, but MMP-9 contributes to plaque instability [33]. At the same time, hypercholesterolemia increases the formation of atherogenic biomolecules, including IL-6, TNF-α, ICAM-1, and VCAM-1 [26]. Their expression is upregulated by nuclear factor-kappa-B (NF-κB) activation during inflammation in atherosclerotic lesions [34]. The stability of atherosclerotic plaques is determined by the presence of inflammatory cells and the thickness of the cap. Plaques with thin caps and abundant immune cells are considered vulnerable. Inflammatory cytokines such as IL-1β and IL-6 worsen and sustain atherosclerosis while attracting additional immune cells [35,36]. To clearly establish the link between immune cells activity and OSA markers, future studies including double staining or cell-specific immune markers would provide more precise cellular characterization.

Many studies highlighted a link between inflammation and cardiometabolic complications in patients with OSA. Fei et al. demonstrated that expression of inflammatory proteins TNF-α, CRP, and IL-6 was higher in obese and hypertensive patients with OSA and that CRP levels were higher in OSA with metabolic syndrome [37]. Peres et al. demonstrated that individuals with ICAM-1 > 816 ng/mL had a significantly higher risk of first cardiovascular events over 8 years after PSG (adjusted HR~3.1 in OSA) [38]. Also, Chetan et al. observed that higher VCAM-1 levels in OSA patients were associated with increased cardiovascular risk, as assessed by SCORE2/SCORE2-OP [39]. Lv et al. showed that higher VCAM-1 was associated with more frequent coronary artery disease in adults with moderate-to-severe OSA [26]. Furthermore, Minoguchi et al. showed that OSA patients with higher IL-6 and TNF-α levels had increased carotid intima-media thickness (IMT) [40]. Further observational studies by Ji et al. and Ciccone et al. confirmed IL-6/TNF-α/CRP correlations with carotid IMT in OSA, suggesting that OSA-related hypoxia and systemic inflammation might be associated with the progression of atherosclerosis and thus increase the risks of cardiovascular and cerebrovascular morbidity in patients with OSA [41,42].

### Limitations of the Study

This study has several limitations that should be acknowledged. First, the study population consisted exclusively of patients with advanced coronary artery disease undergoing CABG, which limits the ability to disentangle the independent effects of OSA from underlying atherosclerosis and associated comorbidities. As a result, the observed associations should not be interpreted as causal relationships. Due to the limited number of female participants, sex-specific analyses could not be reliably performed.

Second, the relatively small sample size reflects the limited availability of intraoperative full-thickness aortic tissue and reduces statistical power, particularly for multivariable analyses and subgroup comparisons. This also constrained our ability to comprehensively adjust for potential confounders such as metabolic factors (e.g., lipid profile, glycemic status, or systemic inflammation markers), which may influence vascular inflammation independently of OSA.

Third, the semi-quantitative immunohistochemical assessment, although widely used, is inherently subject to observer-related variability. To minimize this bias, all samples were evaluated by an experienced pathologist blinded to clinical data, and multiple high-power fields were analyzed; however, the method does not provide fully quantitative protein expression levels.

Fourth, the identification of specific inflammatory cell subtypes (e.g., monocytes/macrophages) was based on morphological assessment without confirmation by cell-specific markers or double immunostaining and should therefore be considered descriptive.

Finally, due to the study design and grouping strategy, we were not able to demonstrate a clear dose–response relationship across all OSA severity categories, and the results are based on comparisons between clinically defined groups rather than continuous correlation analyses.

Despite these limitations, the study provides unique insight into local vascular inflammatory processes directly within the aortic wall, which cannot be captured by circulating biomarkers alone.

## 4. Materials and Methods

### 4.1. Patients and Tissue Samples

We enrolled 62 patients who met the study’s inclusion criteria. Forty-six patients qualified for surgical treatment for stable coronary artery disease and completed the entire protocol. All surgeries involved coronary artery bypass grafting on the beating heart, using the classic approach through a median sternotomy. Depending on the progression of the disease, 1–3 bypasses were performed using the left internal mammary artery (LIMA) and the great saphenous vein (VSM—vena saphena magna). Intraoperatively, a sample of the aorta—a full-wall fragment of the ascending aorta—was taken during venous bypass anastomosis (VSM). Before chest closure, flow in the coronary bypass grafts was measured using a Transition Time Flow Meter (TTFM) probe.

### 4.2. Sleep Study

The diagnosis of OSA was confirmed before surgery using a Home Sleep Apnea Test (HSAT) with the WatchPAT™ (Itamar Medical, Caesarea, Israel) portable sleep apnea diagnostic device. WatchPAT system monitors peripheral arterial tonometry, oximetry, heart rate, actigraphy, body position, and snoring. The Apnea–Hypopnea Index (AHI) was defined as the average number of apneas (complete breathing stops) and hypopneas (partial breathing obstructions) per hour of sleep. OSA severity was classified by the apnea hypopnea index [43] as follows: control, AHI < 5; mild, AHI ≥ 5; moderate, AHI ≥ 15; severe, AHI ≥ 30 events/hour. Sleep studies were performed 1 to 3 days before surgery. For statistical analysis, patients were additionally grouped into control/mild OSA and moderate/severe OSA categories, consistent with commonly used clinical classifications of OSA severity and cardiovascular risk.

### 4.3. Immunohistochemistry

Formalin-fixed, paraffin wax-embedded tissue specimens were cut into 4 μm sections and mounted on SuperFrost^®^Plus adhesion microscope slides (Menzel Gläser) (Menzel Gläser, Braunschweig, Germany). After deparaffinization in xylene and hydration, heat-induced epitope retrieval was carried out by cooking in high-pH EnVision FLEX Target Retrieval solution (Agilent/Dako) (Agilent Technologies, Santa Clara, CA, USA) for 40 min at 97 °C in a water bath (except ICAM—in low pH EnVision FLEX Target Retrieval Solution, Agilent/Dako). At a later stage, the scraps were machine-stained—Autostainer Link 48 (Agilent/Dako)—using a visualization kit for immunohistochemical staining—EnVision Flex + System, High pH (Link) (Agilent/Dako). Santa Cruz Biotechnology (Dallas, TX, USA) mouse monoclonal antibodies were used to test antibody expression: IL-6 (clone E-4) [dilution 1:100], ICAM-1 (clone 15.2) [1:350], VCAM-1 (clone E-10) [1:80], and TNF alfa (clone 52B83) [1:400]. A mouse LINKER (Agilent/Dako) was used to enhance the reaction and optimize IL-6 staining. An experienced pathologist evaluated immunohistochemical staining. For each patient, five randomly selected high-power fields (HPFs) were evaluated within the same aortic tissue section to ensure representative sampling and minimize local variability of staining. The results were interpreted using a light microscope (Axio Observer Z1, Zeiss, Jena, Germany) equipped with Axiovision 4.8 software (Zeiss Microimaging GmbH, Jena, Germany) and the LUMEN200 illumination system (Prior Scientific, Ltd., Jena, Germany).

### 4.4. Evaluation of Immunohistochemistry

The results were presented as the percentage of positive cells within the field of view (FOV), based on an assessment of five randomly selected high-power fields (HPF x200 magnification). The number of positive cells was categorized using the following 4-tiered classification system: 0, negative reaction (less than 10% of stained cells); 1, low expression (11% to 50% of cells stained positive); 2, intermediate expression (51% to 75% of cells stained positive); 3, high expression (over 75% of cells stained positive). To eliminate bias, the pathologist was blinded to the experimental groups.

In addition to the proportion of positive cells, staining intensity was assessed qualitatively during microscopic evaluation by the same blinded pathology consultant. The intensity of immunohistochemical staining was classified as absent, weak, moderate, or strong according to the visual intensity of the chromogenic reaction observed in positively stained inflammatory cells. Staining intensity was considered as part of the overall semi-quantitative pathological interpretation; however, it was not recorded as a separate quantitative variable and therefore was not subjected to independent statistical analysis.

Levels of inflammatory markers measured in aortic wall tissue are presented as semi-quantitative immunohistochemical expression scores based on the proportion of positively stained inflammatory cells. Identification of lymphocyte- and monocyte-like inflammatory cells was based on qualitative morphological assessment by an experienced pathology consultant.

### 4.5. Statistical Analysis

The results are expressed as the mean ± standard deviation. The Mann–Whitney U test was used to evaluate differences between groups, with *p* < 0.05 considered statistically significant. The statistical analyses were performed with SPSS 23.0 software (IBM, Armonk, NY, USA).

Because previous reports have linked ICAM-1, VCAM-1, TNF-α, and IL-6 levels with obesity, we performed proportional odds regression with a cumulative logit link for the four-level ordinal outcome in R using the *polr* function from the MASS package 7.3-65. For each marker, an initial model without BMI was fitted, followed by a model including BMI as a continuous covariate. Nested models were compared using the likelihood-ratio test to assess whether inclusion of BMI improved model fit; as per Ripley B, Venables B. MASS: Support Functions and Datasets for Venables and Ripley’s MASS. R package version 7.3-65.

## 5. Conclusions

In conclusion, this study provides novel evidence that key adhesion molecules like ICAM-1 and VCAM-1 were significantly overexpressed directly within the aortic wall of patients with moderate-to-severe OSA. These findings support the hypothesis that OSA-related intermittent hypoxia may be associated with vascular inflammatory processes related to atherosclerosis.

Importantly, our results highlight that assessment of protein expression in vascular tissue may offer more mechanistic insight than circulating biomarkers, which reflect downstream effects rather than local disease activity. The present findings suggest an association between OSA severity and localized vascular inflammation; however, further studies are needed to clarify the independent contribution of OSA to atherogenesis. Importantly, larger cohorts and quantitative approaches are warranted to confirm these observations and clarify their clinical implications.

## Figures and Tables

**Table 1:** Patient demographic data and home sleep apnea test (HSAT) results in 2 groups of patients divided according to pAHI.

	Control and Mild	Moderate and Severe
Cases n	22	24
Age, years	66.8 ± 12.6	68 ± 11.2
Female/Male, n	4/18	4/20
BMI, kg/m^2^	25.9 ± 2.6	29.8 ± 4.7 *
pAHI, events/h	9.3 ± 5	35.8 ± 16 *
ODI	4 ± 4.5	22.4 ± 15 *
% time SaO2 < 90%	0.1 ± 4.6	2.7 ± 6.2
ESS score	6.5 ± 4.7	8.1 ± 5.1
HbA1c (mmol/mol)	5.9 ± 3.9	8.4 ± 4.09 *
Total cholesterol (mg/dL)	172.4 ± 38.6	198.8 ± 43.7 *
HDL (mg/dL)	46.3 ± 12.5	43.5 ± 10.7
LDL (mg/dL)	115.7 ± 28.6	145.7 ± 28.9 *
Triglycerides (mg/dL)	118.5 ± 23.8	169.5 ± 42.2 *
CRP (mg/dL)	4.2 ± 2.9	6.8 ± 3.2 *
WBC (n × 10^3^/mL)	6.8 ± 3.2	7.9 ± 4.1

BMI, body mass index; pAHI, peripheral arterial tone apnea/hypopnea index; ODI, oxygen desaturation index; ESS, Epworth sleepiness scale; HbA1c, Hemoglobin A1c; HDL, High-Density Lipoprotein; LDL, Low-Density Lipoprotein; CRP, C-reactive protein; WBC, White Blood Cell count. Mean ± SD (standard deviation). * *p* < 0.05 (Mann–Whitney U test).

**Table 2:** Level of inflammatory markers measured in aortic wall of two groups of patients with obstructive sleep apnea: control + mild and moderate + severe.

Control and Mild OSA						Moderate and Severe OSA						
Variable	N	M	SD	Me	Mrang	N	M	SD	Me	Mrang	U	*p*
ICAM-1	22	1.00	0.62	1.00	16.95	24	1.67	0.7	2.00	29.50	120.00	**0.001**
VCAM-1	22	0.09	0.29	0.00	19.09	24	0.46	0.51	0.00	27.54	167.00	**0.006**
TNF-α	22	0.23	0.53	0.00	20.18	24	0.46	0.66	0.00	28.37	169.00	0.159
IL-6	22	0.32	0.72	0.00	20.82	24	0.67	0.87	0.00	25.96	205.00	0.109

N—number of patients; M—mean; SD—standard deviation; Me—median. Mrang = mean rank; U = Mann–Whitney U test; *p* = significance value; levels of inflammatory markers measured in aortic wall tissue are presented as semi-quantitative immunohistochemical expression scores based on the proportion of positively stained inflammatory cells.

## Data Availability

Data is available from the corresponding author of the study upon DTA agreement.
